# Association Testing Strategy for Data from Dense Marker Panels

**DOI:** 10.1371/journal.pone.0080540

**Published:** 2013-11-12

**Authors:** Donghyung Lee, Silviu-Alin Bacanu

**Affiliations:** Department of Psychiatry, Virginia Institute for Psychiatric and Behavioral Genetics, Virginia Commonwealth University, Richmond, Virginia, United States of America; Johns Hopkins University, United States of America

## Abstract

Genome wide association studies have been usually analyzed in a univariate manner. The commonly used univariate tests have one degree of freedom and assume an additive mode of inheritance. The experiment-wise significance of these univariate statistics is obtained by adjusting for multiple testing. Next generation sequencing studies, which assay 10-20 million variants, are beginning to come online. For these studies, the strategy of additive univariate testing and multiple testing adjustment is likely to result in a loss of power due to (1) the substantial multiple testing burden and (2) the possibility of a non-additive causal mode of inheritance. To reduce the power loss we propose: a new method (1) to summarize in a single statistic the strength of the association signals coming from all not-very-rare variants in a linkage disequilibrium block and (2) to incorporate, in any linkage disequilibrium block statistic, the strength of the association signals under multiple modes of inheritance. The proposed linkage disequilibrium block test consists of the sum of squares of nominally significant univariate statistics. We compare the performance of this method to the performance of existing linkage disequilibrium block/gene-based methods. Simulations show that (1) extending methods to combine testing for multiple modes of inheritance leads to substantial power gains, especially for a recessive mode of inheritance, and (2) the proposed method has a good overall performance. Based on simulation results, we provide practical advice on choosing suitable methods for applied analyses.

## Introduction

 Genome-wide association studies (GWASs) have been broadly used to test for association between genetic variants and various phenotypes. These studies have been quite successful in identifying numerous single nucleotide polymorphisms (SNPs) associated with a variety of human traits and diseases [1]. So far, GWASs have been commonly analyzed univariately using one degree of freedom (df) tests assuming an additive mode of inheritance [2–4]. The experiment-wise significance of the univariate statistics was assessed using a Bonferroni adjustment [5,6] or a permutation procedure [7–9]. While this approach was reasonably successful for GWAS, the field is moving away from this paradigm towards whole genome sequencing. When compared to GWAS, variant panels for sequencing studies (1) are substantially denser and (2) have different patterns of linkage disequilibrium (LD). Consequently, for these studies it is not clear if the most desirable approach is still a univariate testing for an additive mode of inheritance followed by the adjustment of the statistics for the large number of tests. 

 Intuitively, a test for association between phenotype and genotype achieves optimal power when the assumed mode of inheritance matches the underlying one. However, the underlying mode of inheritance is usually unknown in practice and an incorrect choice for it may cause a substantial power loss. Various strategies to minimize the effect of the possible model misspecification have been studied and developed [10–17]. Among these, one simple strategy is to test other modes of inheritance, e.g. dominant and recessive, in addition to the commonly used additive mode of inheritance [16,17]. This approach involves testing for three different modes of inheritance, i.e. additive (A), dominant (D) and recessive (R), and adjusting the lowest p-values for multiple testing. For brevity, the combination of testing for the three modes of inheritance will be henceforth denoted as ADR. Because the ADR paradigm is not in widespread use yet, it is of interest to estimate the performance improvement when applied to methods which were initially developed assuming an additive mode of inheritance.

 To control the rate of false positives in GWAS analyses, the statistical significance of univariate p-values is adjusted for around a million univariate tests. With the advent of next generation sequencing, for univariate analyses, the number of tests will increase dramatically when compared to GWAS. Summary data from 1000 Genomes Project suggests that sequencing studies consisting of subjects from any main ethnic regions, i.e. Europe, East Asia, South Asia, Africa and the Americas, will result in the typing of at least 5 million SNPs having a minor allele frequency (MAF) above 5% [18]. For larger sequencing studies, if we assume that all SNPs with MAF > 0.5% are analyzed individually, the number of tests increases to more than 10 million for Caucasian and Asian cohorts and approaches 20 million for African cohorts. Consequently, univariate approaches entail an even more substantial multiple testing adjustment burden for sequencing studies. It is conceivable that using multilocus approaches, e.g. by summarizing the association of multiple SNPs simultaneously, opens the possibility of decreasing the multiple testing burden and, thus, increasing the power of detection for association signals. 

 To minimize the power penalty due to multiple testing adjustment, researchers proposed to analyze simultaneously all SNPs in a biological functional block of interest, e.g. a gene [19]. However, this approach might yield low power due to the large number of df. Subsequently, researchers proposed methods to decrease the number of df, and thus increase power, by (1) summarizing the LD information mainly from low-frequency SNP variation in an LD block [20], (2) using data-adaptive sum of squared scores (aSUM) [21], (3) summarizing the LD information by the first few principal components (PC) [22,23], (4) combining the Simes p-value of all univariate p-values in a gene with the p-value associated with the first few principal components of tests in the gene(S-PC) [24] and (5) combining individual SNP-based variance component score statistics (SKAT) [25].

 Recently, two fast non-regression based multilocus methods were proposed for gene-based analysis. The first method, denoted as VErsatile Gene-based Association Study (VEGAS), summarizes the association signals in a gene using the sum of squares (SS) or the minimum p-value (minP) of univariate statistics in the gene [26]. Instead of performing permutations, VEGAS simulates multivariate normal variables for a rapid assessment of the asymptotic null distribution of summary statistics. The second method is an improved Simes procedure for association studies, denoted as Gene-based Association Test using Extended Simes (GATES) [27]. GATES is an extension to a technique proposed initially by Cheverud [28] to determine the effective number of independent tests in a region of interest. (For more background regarding this type of methods, see 28–32.) Because VEGAS-SS (V-SS), VEGAS-minP (V-minP) and GATES do not use permutations, they are faster than permutation based multilocus methods. 

 While the gene is commonly considered as the biological functional unit, it might not be the best unit for statistical analysis. First, since not all parts of a gene are equally important functionally, it would be more powerful to analyze separately SNPs in regions with important functions such as gene promoter regions, which are known to be involved in initiating and regulating the transcription process [33,34]. Second, the association signal from a causal SNP, e.g. from the promoter region, is diffused only among SNPs in the same LD block [20]. Thus, using as the unit of analysis a gene containing multiple LD blocks might increase the noise and reduce overall statistical power to detect an association signal. An alternative unit of statistical analysis might be a LD block (e.g. SNPs with  D ′ near 1). 

 Unlike GWAS, which are based on a tag SNP approach, whole genome sequencing studies assay most genetic variants in the genome. Consequently, the typed variation in these studies will likely form a large number of LD blocks, each block having a large number of SNPs. Given the number and size of these blocks, it is conceivable that approaches analyzing simultaneously all SNPs in a LD block might be of great importance for a successful investigation of sequencing studies. Although the partition of the genome into LD block requires a substantial computing time, once they are computed for the main ethnic groups, these blocks can be reused for future analyses. While the LD block analysis approach was investigated before [17], these findings need to be updated for the present-day technological and methodological environment, i.e. increased variant density and the new developments in gene based methods. 

 In this paper, we attempt to develop/identify the most powerful methods to detect association signals in a LD block. To achieve this goal, we (1) propose a simple statistic consisting of the sum of the squares of significant univariate tests in a LD block, (2) use two simulation experiments to assess the performance of the proposed method and competing methods a) with or b) without an ADR extension and (3) provide practical recommendations based on simulation results.

## Materials and Methods

### Methods

 Assume that we are interested in assessing the association between a binary phenotype, *Y*, and *m* SNPs in a LD block using a case-control cohort consisting of *n* cases and *n* controls. For the *i* ^th^ subject, let $Y_{i}=\{\begin{array}{cc} 1 & \text{if }i^{\text{th}}\text{ subject is a case}\\ 0 & \text{if }i^{\text{th}}\text{ subject is a control} \end{array}$, *i*= 1,…,2*n*, be the phenotype and ***G***_*A*,*i*⋅_=(*G*_*A*,*i*1_,…,*G*_*A*,*im*_) be the additively coded genotypes for the *m* SNPs in the LD block, i.e. the number of reference alleles. To test for a dominant mode of inheritance, we denote the indicators for the heterozygote and the reference allele homozygote as ***G***_*D*,*i*⋅_=(*G*_*D*,*i*1_,...,*G*_*D*,*im*_). To test for a recessive mode of inheritance, let the indicators of reference allele homozygote be ***G***_*R*,*i*⋅_=(*G*_*R*,*i*1_,...,*G*_*R*,*im*_). For each mode of inheritance, we can obtain the vector of normally distributed test statistics by regressing the phenotype on the relevant genotype vector. Assume that the vectors of test statistics corresponding to the additive, dominant and recessive modes of inheritance are ***Z***_*A*_=(*Z*_*A*,1_,...,*Z*_*A*,*m*_), ***Z***_*D*_=(*Z*_*D*,1_,...,*Z*_*D*,*m*_) and ***Z***_*R*_=(*Z*_*R*,1_,...,*Z*_*R*,*m*_), respectively. Based on these statistics, we can compute p-value vectors for the three modes of inheritance as ***p***_*A*_=(*p*_*A*,1_,...,*p*_*A*,*m*_), ***p***_*D*_=(*p*_*D*,1_,...,*p*_*D*,*m*_) and ***p***_*R*_=(*p*_*R*,1_,...,*p*_*R*,*m*_). 

 Certain gene based approaches, such as V-SS, sum the squares of all univariate statistics in a LD block. However, this approach might lose power by including SNPs with weak association signals, which only add noise to the test statistic. V-minP also might suffer some statistical power loss by using only the most significant statistic. To avoid such a loss of power we propose a new statistical test consisting of the sum of squared statistics exceeding a threshold, i.e.$\sum_{j=1}^{m}Z_{A,j}^{2}I(Z_{A,j}^{2}\geq t)$, where *t*>0 is a reasonably high threshold. We denote this test statistic as the sum of square above a threshold (SS-T). The statistical significance of SS-T can be assessed in a manner similar to V-SS and V-minP, i.e. via multivariate normal simulations based on the LD pattern in the block.

 We compare the performance of the proposed test (SS-T), to the performance of various association tests developed mainly for an additive mode of inheritance and, where possible, their ADR extensions. As association tests, we include in our simulation studies Bonferroni, Simes [35], GATES [27], V-SS/V-minP [26], PC [22,23], S-PC [24], SKAT [25] and aSUM [21]. In our comparisons we also include the ADR extensions for SS-T (ADR SS-T), Bonferroni (ADR Bonferroni), Simes (ADR Simes), GATES (ADR GATES), V-SS (ADR V-SS), V-minP (ADR V-minP), PC (ADR PC) and S-PC (ADR S-PC). ADR extensions for SKAT and aSUM are not attempted because the additive coding for genotype is implicit in the software implementing these methods. 

 Bonferroni procedure summarizes the *m* univariate p-values in a LD-block p-value defined as min_*j*_(*m p*_*A*,*j*_), *j*=1,…,*m*. Simes method alleviates the conservativeness of Bonferroni adjustment by using $\min_{j}(\frac{m\;p_{A,j}}{j})$, *j*=1,…,*m*, as the block p-value [35]. GATES method enhances the power of Simes by using an effective number of tests approach. The p-value of GATES method is obtained as $\min_{j}(\frac{m_{eff}\;p_{A,(j)}}{m_{eff(j)}})$, *j*=1,…,*m*, where *m*_*eff*_ is the effective number of tests of all p-values (*p*_*A*,1_,...,*p*_*A*,*m*_) and *m*_*eff*(*j*)_ is the effective number of tests computed from the *j* smallest p-values (*p*_*A*,(1)_,...,*p*_*A*,(*j*)_) [27]. V-SS and V-minP compute the sum of squares, $\sum_{j=1}^{m}Z_{A,j}^{2}$, and minimum p-value (which is equivalent to $\max_{j}(Z_{A,j}^{2})$) of the univariate statistics and assess their significance by simulating their null distributions based on the multivariate normal distribution [26]. The test statistic for the PC method, *Q*_*k*_, is defined as the sum of squares of the first *k* PC statistics, denoted as *U*_1_,…,*U*_*k*_, of the genotype correlation matrix. The *j* ^th^ PC statistic is defined as $U_{j}=v_{j}\cdot Z_{A}/\sqrt{\lambda_{j}}$, where *v*_*j*_ and *λ*_*j*_ are the *j* ^th^ eigenvector and eigenvalue of the correlation matrix of genotype data *G*_*A*,⋅⋅_[22,23]. Under the null hypothesis, of no association between genotype and phenotype, *Q*_*k*_ is distributed as a *χ* ^2^ with *k* df. In our comparison studies, we consider the first three PC statistics, i.e. *k*=3. The p-value for S-PC is obtained by performing a Simes correction on the p-values generated from (i) a Simes procedure applied on *p*_*A*_ and (ii) the above PC method [24]. 

 The ADR extension for methods in the previous paragraph can be achieved similarly by substituting: (i) 3*m* for *m* as the number of tests in the LD block, (ii) ADR p-values (***p***_*ADR*_=(***p***_*A*_,***p***_*D*_,***p***_*R*_)) for p-values assuming an additive mode of inheritance (*p*_*A*_) and (iii) ADR univariate statistics (***Z***_*ADR*_=(***Z***_*A*_,***Z***_*D*_,***Z***_*R*_)) for univariate statistics assuming an additive mode of inheritance (***Z***_*A*_). While the three tests (A, D and R) are not independent, both within and between SNPs, given that the power loss is small [17], we conservatively use 3*m* as the number of tests for the ADR extension for Bonferroni and related methods.

 We implemented in R all methods tested in this paper, with the exception of SKAT and aSUM. SKAT statistics were obtained using SKAT 0.63 R package with a linear kernel and the default options. For aSUM, we used its implementation from AssotesteR 0.1-1 R package (http://www.gastonsanchez.com/assotester) with the default options. 

### Simulations

 We employ two extensive simulation experiments to generate genotype-phenotype data sets that we subsequently use to assess the performance of relevant methods and their ADR-extensions. The first simulation experiment generates artificial LD-patterns (see Tables S1-S3) and the second experiment is based on LD-patterns from a real data set (see Tables 1 and 2). 

**Table 1:** Number of cases/controls (*n*), relative risk of heterozygote to non-risk allele homozygote (*R*_1_) and relative risk of risk allele homozygote to non-risk allele homozygote (*R*_2_) used at each simulation setting under the single causal variant scenario of Experiment II.

		Genetic Model		
Number of causal variants (*k*)	Additive		Dominant	Recessive
1	1,000 (1.3, 1.6)		1,000 (1.5, 1.5)	1,000 (1, 3)

Within each cell, the settings are presented as *n* (*R*_1_, *R*_2_).

**Table 2:** Number of cases/controls (*n*) and effect size (*δ*) of any causal allele used at each simulation setting under the two and five non-interacting causal variant scenario of Experiment II.

		Genetic Model		
Number of causal variants (*k*)	Additive		Dominant	Recessive
2	1,000 (0.005)		1,000 (0.008)	1,000 (0.03)
5	1,000 (0.002)		1,000 (0.003)	1,000 (0.01)

Within each cell, the settings are presented as *n* (*δ*).

 To efficiently simulate a large number of correlated SNPs in a LD block, the first simulation experiment (Experiment I) employs the polychoric correlation (PCC) as a measure of LD [17,36]. The advantage of PCC over other LD measures is that it is directly applicable to simulating a large number of markers in LD regardless of their reference allele frequencies (RAFs) [17]. The simulation of a study cohort is achieved using a four-step process: i) simulate latent multivariate normal variables, ii) discretize the latent variables to obtain genotypes, iii) simulate the phenotype, i.e. case or control, for the simulated genotype vector and iv) accept cases and controls until achieving the required sample size. In this experiment, we investigate three settings for the number of causal variants (*k*): i) null hypothesis, i.e. no causal variant (*k*=0), ii) a single causal variant (*k*=1) and iii) two non-interacting causal varaints (*k*=2). We present the simulation process in more detail under Supporting Information (see Methods S1). 

 The second simulation experiment (Experiment II) is based on 200 randomly chosen 250Kb genetic regions from UK10K (www.uk10k.org) reference data set. LD blocks are inferred by performing a hierarchical clustering analysis with an average link on SNP genotypes in each selected region. To match one of the settings from the first experiment, the LD blocks are defined using a PCC^2^ threshold of 0.64. The causal LD block and the causal variant(s) within it are chosen randomly and, thus, their RAFs are not fixed. When compared to Experiment I, to increase robustness of the findings, we add to this simulation experiment a five non-interacting causal variant scenario (*k*=5). Otherwise, the simulations for this experiment follow the conceptual flow of the first experiment.

 To examine the effect of the causal model on the power of various methods, we simulated case-control cohorts under three underlying modes of inheritance, i.e. additive, dominant and recessive (see Tables S2 and S3 for Experiment I and Tables 1 and 2 for Experiment II). Under *k* causal variant scenario (*k*>1), the underlying genetic modes of inheritance of all *k* casual SNPs are assumed to be identical (for more details see Methods S1). Each case-control sample consists of 1,000 cases and 1,000 controls for most settings (see Tables S2 and S3 for Experiment I and Tables 1 and 2 for Experiment II). To attain the 50% average power target, for Experiment I the genotype penetrances and the sample sizes vary with the mode of inheritance and the causal allele frequency (see Tables S2 and S3).

 For both experiments, we assume a binary trait of prevalence *K*=0.05 and assess the empirical size of the test and power for tested methods at a type I error of0.05. We obtain the size and power of each method by simulating 500 replicates at each setting. For Monte-Carlo simulation/permutation based methods, such as V-SS, V-minP, SS-T and aSUM, we performed 500 and 1,000 simulations under Experiment I and II, respectively. 

## Results

**Figure 1:**
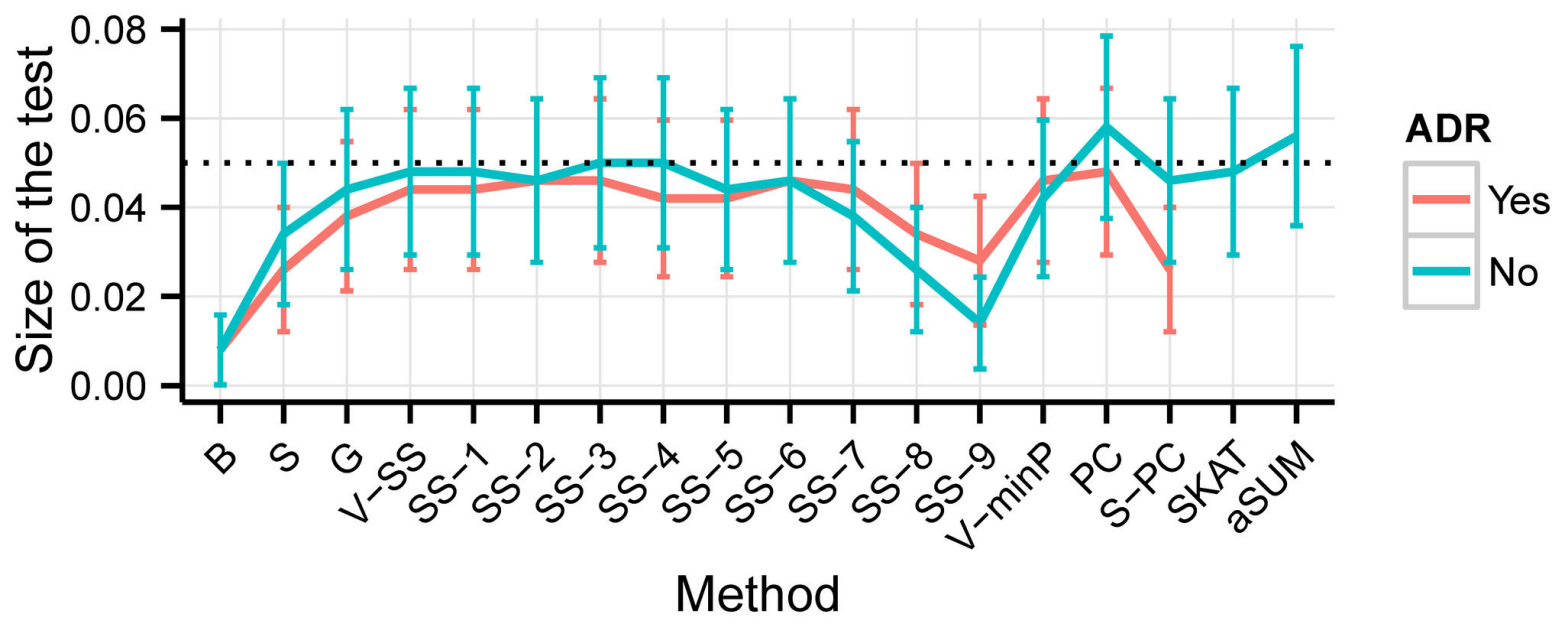
Empirical size of the test under Experiment II as a function of the method type and the ADR adjustment status. The nominal type I error rate is *α*=0.05. The bars represent the 95% confidence interval for the size of test. Abbreviations for methods are as follows: B - Bonferroni, S - Simes, G - GATES, V-SS - VEGAS-SS, SS-x - SS-T with *x*=1,...,9, V-minP - VEGAS-minP, PC - principal component method, S-PC - Simes adjustment of Simes and PC methods, SKAT - sequence kernel association test, aSUM - data-adaptive sum test.

 For Experiment I, under the null hypothesis (of no association between SNP genotypes and trait) some tests (V-SS, V-minP, SS-T and aSUM) using Monte-Carlo simulation/permutation to assess the significance of the test statistic seem to have slightly (albeit not-statistically significant) inflated size of the test (Figure S1). However, when we increase to 1000 the number of replications for Experiment II, all tested methods control the size of the test at or below the nominal type I error (Figures 1). As a general rule, ADR extensions tend to make most methods more conservative. Simpler adjustments for multiple comparisons, e.g. Bonferroni and Simes, their ADR extensions and SS-T are the most conservative. 

**Figure 2:**
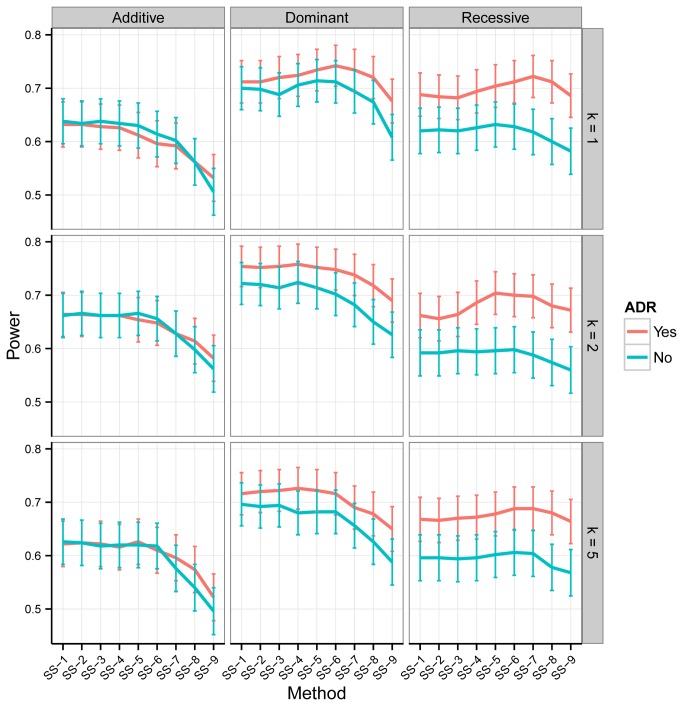
Empirical power of SS-T methods under Experiment II as a function of the mode of inheritance, the number of causal variants in a LD block (*k*) and the ADR adjustment status. The nominal type I error rate is *α*=0.05. The bars represent the 95% confidence interval for the power of test. See Figure 1 for background and abbreviations.

 To find a reasonable threshold value for SS-T we use the results of Experiment I and II, which are summarized in Figures S2-S7 and Figure 2, respectively. Under the first experiment, as the threshold value increases from 1 to 9, power tends to rise. This behavior is more apparent especially when 1) the mode of inheritance is recessive, 2) the tests are ADR-adjusted, 3) the size of the LD block is small and 4) the polychoric correlation between the genotypes in the LD block is high. Under Experiment II, where we use realistic LD blocks from UK10K data set, power decreases with an increase in threshold values. Though under the dominant and recessive modes of inheritance power tends to slightly increase as the threshold value increases from 4 to 6, the improvement in power is marginal compared to that observed under Experiment I. On the basis of the results from both comparisons, we deem SS-6 to be close to optimal power-wise and, henceforth we present only its performance. 

 Under Experiment I conditions, the single causal variant simulations show differences in power between the ADR adjusted methods and the non-ADR-adjusted methods (Figures S8-S10). When the underlying modes of inheritance are additive or dominant, the ADR-adjustment causes a small power loss averaging 4.9% (see "Additive" and "Dominant" panels in the above mentioned figures). However, for a recessive mode of inheritance, the adjustment considerably improves the power to detect the association signal for most settings. The average ADR adjustment power gain under a recessive mode of inheritance is around 34.3%, ranging from 6.9% for PC to 51.8% for V-minP. Under a dual causal variant scenario, the differences in power between methods and their ADR extensions are similar to the single causal variant scenario (Figures S11-S13). ADR adjustment results in a 5.3% decrease in average power for non-recessive models and a 30% increase in average power for a recessive mode of inheritance. The average power gain of the methods under a recessive mode of inheritance ranges from 8.9% for V-SS to 40.5% for V-minP. The power gain for both (single and dual) causal variant scenarios under a recessive mode of inheritance appears to increase with i) an increase in the polychoric correlation between SNPs in the LD block, ii) a decrease in the size of the LD block and iii) a decrease in causal allele frequency (CAF). 

**Figure 3:**
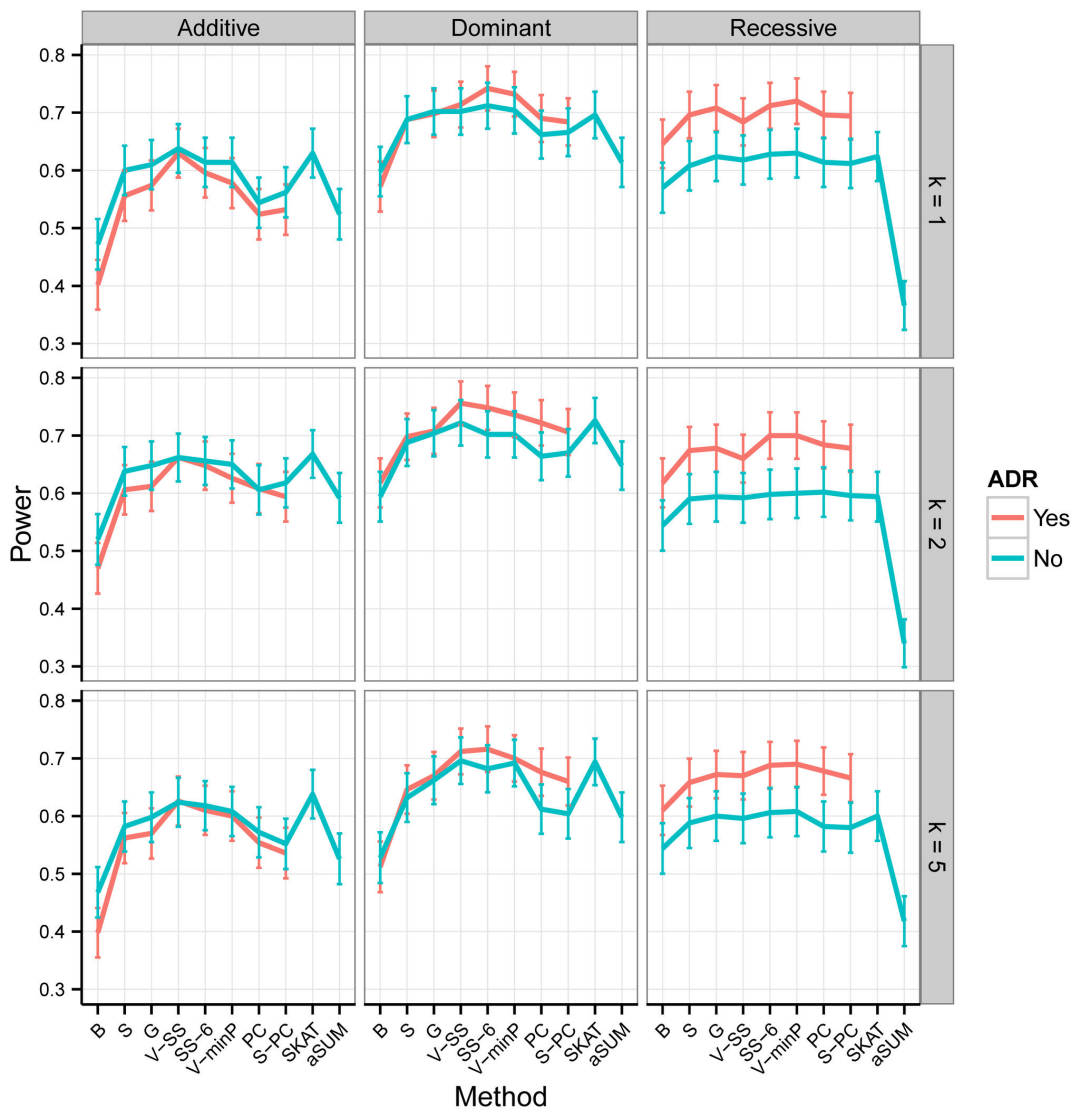
Empirical power of the main methods under Experiment II as a function of the mode of inheritance, the number of causal variants in a LD block (*k*) and the ADR adjustment status. The nominal type I error rate is *α*=0.05. The bars represent the 95% confidence interval for the power of test. See Figure 1 for background and abbreviations.

 Besides the difference in power between methods and their ADR extensions, it is of interest to establish which methods perform better under varying scenarios. For the first experiment, aSUM tends to have the highest power under additive/dominant modes of inheritance and it outperforms, sometimes considerably, the next best performing group (V-minP, GATES, SS-6 and their ADR extensions) (Figures S8-S13). Under additive/dominant modes of inheritance, as the CAF increases the difference in power between methods gradually lessens while the rank of each method tends to be maintained. For a recessive mode of inheritance, ADR V-minP and ADR GATES perform best overall and are followed by ADR Bonferroni, ADR Simes, ADR S-PC and ADR SS-6. These methods substantially outperform all non-ADR-adjusted methods, ADR V-SS and ADR PC. As CAF increases, under a recessive mode of inheritance, the performance of ADR SS-6 approaches that of the best performers (ADR V-minP and ADR GATES). For Experiment II, under the additive mode of inheritance SKAT, V-SS, V-minP and SS-6 performed best (Figure 3). Under the non-additive mode of inheritance, ADR SS-6, ADR V-SS and ADR V-minP tend to have highest power. While, as we observed from Experiment I, under the additive model ADR adjustment results in slight power loss, under the dominant model it leads to slight power gain. Under the recessive model, ADR adjustment improves power significantly. When compared to Experiment I, SKAT performs much better under Experiment II conditions. On the other hand, aSUM performs rather poorly when compared to its excellent performance observed in the first simulation experiment. Even more, under a recessive mode of inheritance, aSUM had the lowest power in the second experiment. 

## Discussion

 When compared to GWAS, whole genome sequencing studies assay tens of millions of genetic variants in human genome. Due to their large number, a univariate analysis of these variants involves a substantial multiple testing burden. To avoid such a burden, we propose a new method to summarize in a single statistic the association between phenotype and the genotypes of not-very-rare SNPs, i.e. those which can be reasonably analyzed individually, in a LD block. We use two simulation experiments to compare the performances of i) the proposed method, ii) competing methods, e.g. methods used for gene-based analyses and iii) the extensions of the previously mentioned methods which combine information from multiple modes of inheritance. The results of these simulations are helpful in identifying methods delivering close to optimal power when used to analyze data coming from sequencing studies. 

 One important conclusion of this paper is that, even for denser marker panels, when the true mode of inheritance is unknown, combining additive, dominant and recessive modes of inheritance together (i.e. ADR adjustment) is a suitable strategy to minimize the power loss caused by the model misspecification. The power gain of ADR adjustments over their non-ADR adjusted counterparts is due to the considerable improvement in the overall performance of the methods when the underlying mode of inheritance is recessive. The power gain of ADR extensions increases with an increase in LD between SNPs and a decrease in causal allele frequency. This behavior is consistent with findings for GWAS panels [16,17]. 

 Under the theoretical experiment (Experiment I) with additive and dominant modes of inheritance, for the rarer causal allele frequencies range we assumed, aSUM performs best across all configurations closely followed by V-minP, GATES, SS-6 and their ADR extensions. Under a recessive mode of inheritance, the most powerful methods are ADR V-minP and ADR GATES, which are closely followed by ADR Bonferroni, ADR Simes, ADR S-PC and ADR SS-6. Probably because it assumes additively coded genotypes, aSUM yields low power under a recessive mode of inheritance. However, the realistic experiment (Experiment II) shows that SKAT and V-SS perform well. We believe that the relative difference in both experiments is due to the fact that the first experiment is biased towards common tag SNP panels, whereas the second simulation experiment is geared towards denser, sequencing SNP panel. 

 Based on our simulation results, if the disease associated SNPs are highly likely to be acting additively (multiplicatively) or dominantly, for data coming from sequencing panels, we recommend the use of SKAT, ADR V-SS, ADR V-minP, and ADR SS-T. However, researchers rarely know the mode of inheritance for a variant. When no prior information regarding the underlying mode of inheritance is available, we recommend the use of methods with good performance across all modes of inheritance and all simulation experiments, i.e. ADR V-SS, ADR V-minP, ADR GATES and ADR SS-T. However, we believe that if the authors of SKAT and aSUM implement ADR adjustments in their software, these methods would become desirable tools for the analyses of SNPs in LD blocks regardless of the underlying mode of inheritance. 

## Supporting Information

Figure S1 **The size of the test under Experiment I as a function of the method type, the number of SNPs (*m*), the polychoric correlation (ρ) between the genotypes of SNPs in the LD block and the ADR adjustment status.** The nominal type I error rate is *α*=0.05. Abbreviations for methods are as follows: B - Bonferroni, S - Simes, G - GATES, V-SS - VEGAS-SS, SS-x - SS-T with *x*=1,...,9, V-minP - VEGAS-minP, PC - principal component method, S-PC - Simes adjustment of Simes and PC methods, SKAT - sequence kernel association test, aSUM - data-adaptive sum test.
(TIF)

Figure S2 **Empirical power of SS-T methods for the single causal variant scenario (*k*=1) under Experiment I as a function of the mode of inheritance (panels), the number of SNPs in the LD block (*m*), the polychoric correlation between the genotypes of SNPs in the LD block (*ρ*) and the ADR adjustment status.** The causal allele frequency is *p*_*d*_=0.01 and the nominal type I error rate is *α*=0.05. See Figure S1 for background and abbreviations.
(TIF)

Figure S3 **Empirical power of SS-T methods for the single causal variant scenario (*k*=1) under Experiment I as a function of the mode of inheritance (panels), the number of SNPs in the LD block (*m*), the polychoric correlation between the genotypes of SNPs in the LD block (*ρ*) and the ADR adjustment status.** The causal allele frequency is *p*_*d*_=0.05 and the nominal type I error rate is *α*=0.05. See Figure S1 for background and abbreviations.
(TIF)

Figure S4 **Empirical power of SS-T methods for the single causal variant scenario (*k*=1) under Experiment I as a function of the mode of inheritance (panels), the number of SNPs in the LD block (*m*), the polychoric correlation between the genotypes of SNPs in the LD block (*ρ*) and the ADR adjustment status.** The causal allele frequency is *p*_*d*_=0.10 and the nominal type I error rate is *α*=0.05. See Figure S1 for background and abbreviations.
(TIF)

Figure S5 **Empirical power of SS-T methods for the dual non-interacting causal variant scenario (*k*=2) under Experiment I as a function of the mode of inheritance (panels), the number of SNPs in the LD block (*m*), the polychoric correlation between the genotypes of SNPs in the LD block (*ρ*) and the ADR adjustment status.** The causal allele frequency is *p*_*d*_=0.01 and the nominal type I error rate is *α*=0.05. See Figure S1 for background and abbreviations.
(TIF)

Figure S6 **Empirical power of SS-T methods for the dual non-interacting causal variant scenario (*k*=2) under Experiment I as a function of the mode of inheritance (panels), the number of SNPs in the LD block (*m*), the polychoric correlation between the genotypes of SNPs in the LD block (*ρ*) and the ADR adjustment status.** The causal allele frequency is *p*_*d*_=0.05 and the nominal type I error rate is *α*=0.05. See Figure S1 for background and abbreviations.
(TIF)

Figure S7 **Empirical power of SS-T methods for the dual non-interacting causal variant scenario (*k*=2) under Experiment I as a function of the mode of inheritance (panels), the number of SNPs in the LD block (*m*), the polychoric correlation between the genotypes of SNPs in the LD block (*ρ*) and the ADR adjustment status.** The causal allele frequency is *p*_*d*_=0.10 and the nominal type I error rate is *α*=0.05. See Figure S1 for background and abbreviations.
(TIF)

Figure S8 **Empirical power of the main methods for the single causal variant scenario (*k*=1) under Experiment I as a function of the mode of inheritance (panels), the number of SNPs in the LD block (*m*), the polychoric correlation between the genotypes of SNPs in the LD block (*ρ*) and the ADR adjustment status.** The causal allele frequency is *p*_*d*_=0.01 and the nominal type I error rate is *α*=0.05. See Figure S1 for background and abbreviations.
(TIF)

Figure S9 **Empirical power of the main methods for the single causal variant scenario (*k*=1) under Experiment I as a function of the mode of inheritance (panels), the number of SNPs in the LD block (*m*), the polychoric correlation between the genotypes of SNPs in the LD block (*ρ*) and the ADR adjustment status.** The causal allele frequency is *p*_*d*_=0.05 and the nominal type I error rate is *α*=0.05. See Figure S1 for background and abbreviations.
(TIF)

Figure S10 **Empirical power of the main methods for the single causal variant scenario (*k*=1) under Experiment I as a function of the mode of inheritance (panels), the number of SNPs in the LD block (*m*), the polychoric correlation between the genotypes of SNPs in the LD block (*ρ*) and the ADR adjustment status.** The causal allele frequency is *p*_*d*_=0.10 and the nominal type I error rate is *α*=0.05. See Figure S1 for background and abbreviations.
(TIF)

Figure S11 **Empirical power of the main methods for the dual non-interacting causal variant scenario (*k*=2) under Experiment I as a function of the mode of inheritance (panels), the number of SNPs in the LD block (*m*), the polychoric correlation between the genotypes of SNPs in the LD block (*ρ*) and the ADR adjustment status.** The causal allele frequency is *p*_*d*_=0.01 and the nominal type I error rate is *α*=0.05. See Figure S1 for background and abbreviations.
(TIF)

Figure S12 **Empirical power of the main methods for the dual non-interacting causal variant scenario (*k*=2) under Experiment I as a function of the mode of inheritance (panels), the number of SNPs in the LD block (*m*), the polychoric correlation between the genotypes of SNPs in the LD block (*ρ*) and the ADR adjustment status.** The causal allele frequency is *p*_*d*_=0.05 and the nominal type I error rate is *α*=0.05. See Figure S1 for background and abbreviations.
(TIF)

Figure S13 **Empirical power of the main methods for the dual non-interacting causal variant scenario (*k*=2) under Experiment I as a function of the mode of inheritance (panels), the number of SNPs in the LD block (*m*), the polychoric correlation between the genotypes of SNPs in the LD block (*ρ*) and the ADR adjustment status.** The causal allele frequency is *p*_*d*_=0.10 and the nominal type I error rate is *α*=0.05. See Figure S1 for background and abbreviations.
(TIF)

methods S1 **Simulation of genotype-phenotype data.**
(DOC)

Table S1 **Settings used in Experiment I.**
(DOC)

Table S2 **Number of cases/controls (*n*), relative risk of heterozygote to non-risk allele homozygote (*R*_1_) and relative risk of risk allele homozygote to non-risk allele homozygote (*R*_2_) used at each simulation setting under the single causal variant scenario (*k*=1) of Experiment I.** Within each cell, the settings are presented as *n* (*R*_1_, *R*_2_).
(DOC)

Table S3 **Number of cases/controls (*n*) and effect size (*δ*) of any causal allele used at each simulation setting under the two non-interacting causal variant scenario (*k*=2) of Experiment I.** Within each cell, the settings are presented as *n* (*δ*).
(DOC)
