# Supplementary figures and images for: Association Testing Strategy for Data from Dense Marker Panels
Source: PLoS One. 2013 Nov 12;8(11):e80540. doi: 10.1371/journal.pone.0080540 (PMC3827222; doi:10.1371/journal.pone.0080540)

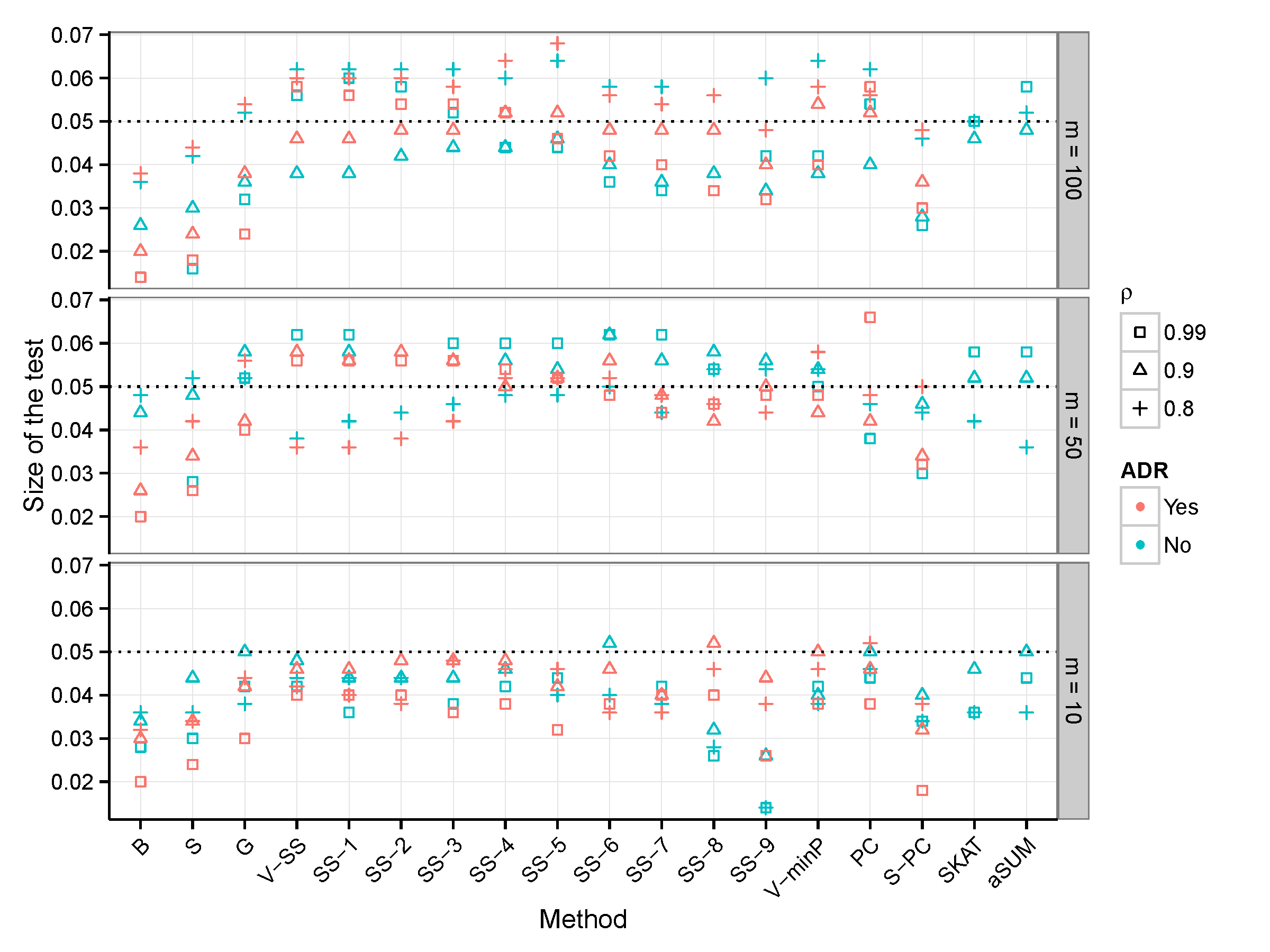

Supplement: Figure S1 — The size of the test under Experiment I as a function of the method type, the number of SNPs (m), the polychoric correlation (ρ) between the genotypes of SNPs in the LD block and the ADR adjustment status. The nominal type I error rate is α=0.05. Abbreviations for methods are as follows: B - Bonferroni, S - Simes, G - GATES, V-SS - VEGAS-SS, SS-x - SS-T with x=1,...,9, V-minP - VEGAS-minP, PC - principal component method, S-PC - Simes adjustment of Simes and PC methods, SKAT - sequence kernel association test, aSUM - data-adaptive sum test. (TIF) [file pone.0080540.s001.tif]

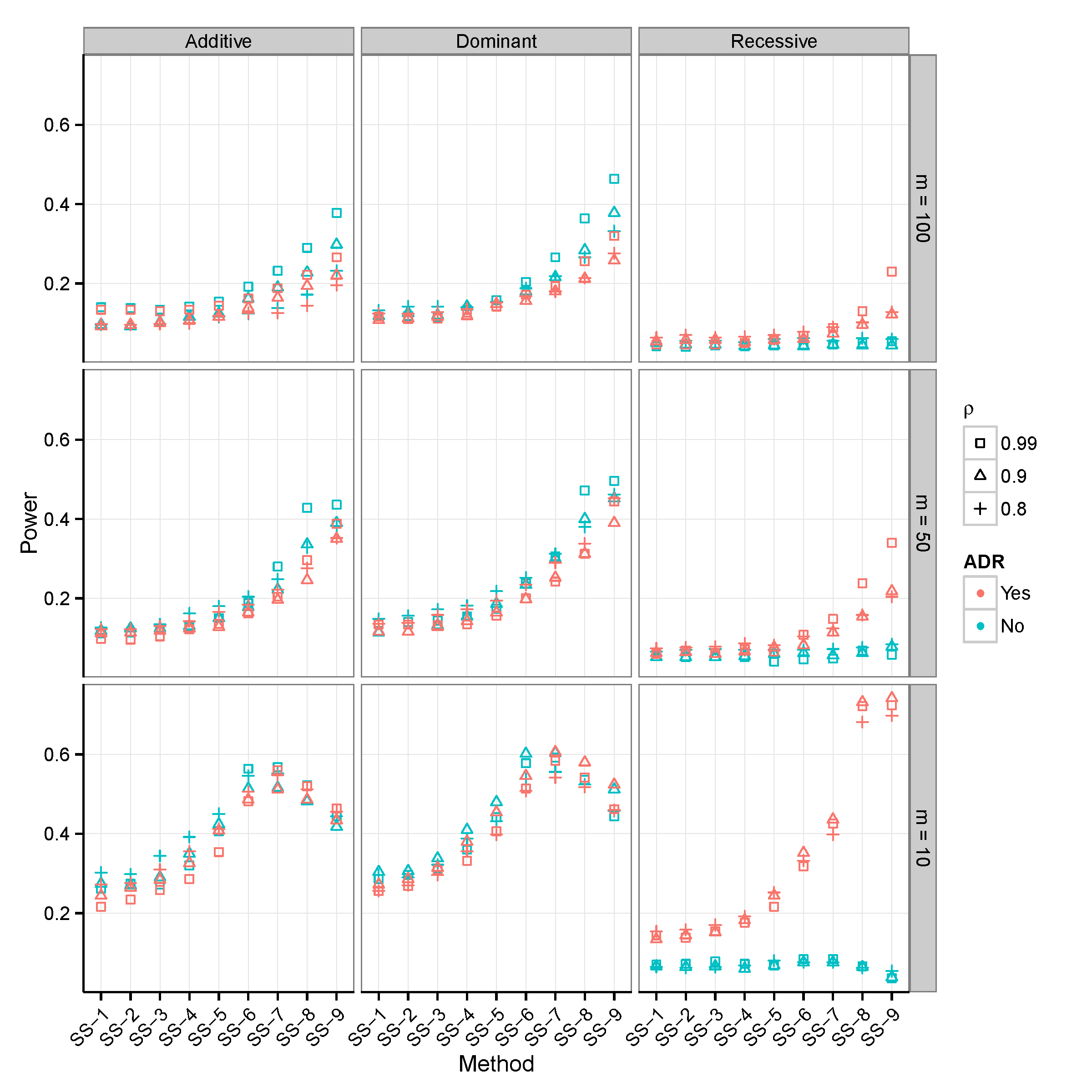

Supplement: Figure S2 — Empirical power of SS-T methods for the single causal variant scenario (k=1) under Experiment I as a function of the mode of inheritance (panels), the number of SNPs in the LD block (m), the polychoric correlation between the genotypes of SNPs in the LD block (ρ) and the ADR adjustment status. The causal allele frequency is p d=0.01 and the nominal type I error rate is α=0.05. See Figure S1 for background and abbreviations. (TIF) [file pone.0080540.s002.tif]

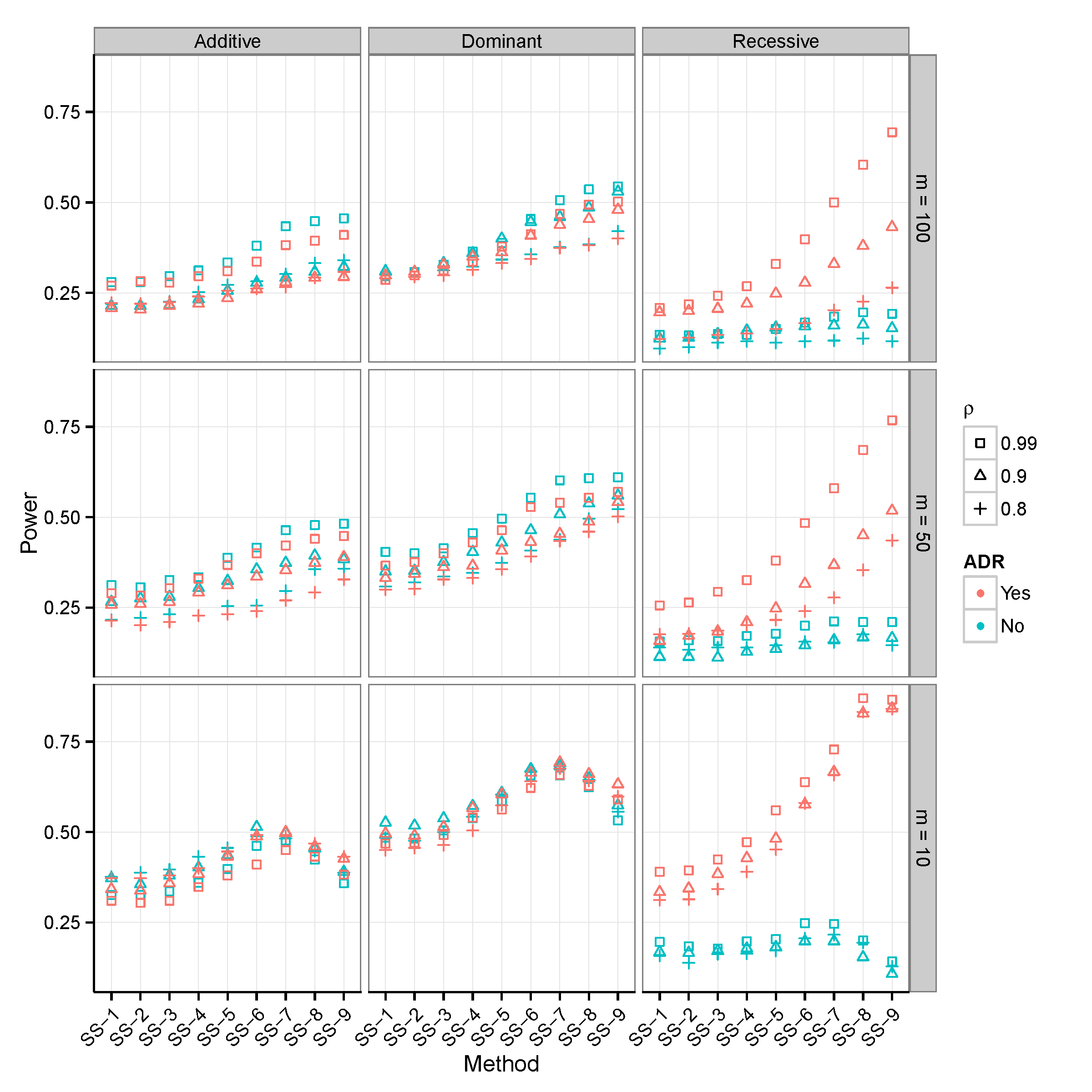

Supplement: Figure S3 — Empirical power of SS-T methods for the single causal variant scenario (k=1) under Experiment I as a function of the mode of inheritance (panels), the number of SNPs in the LD block (m), the polychoric correlation between the genotypes of SNPs in the LD block (ρ) and the ADR adjustment status. The causal allele frequency is p d=0.05 and the nominal type I error rate is α=0.05. See Figure S1 for background and abbreviations. (TIF) [file pone.0080540.s003.tif]

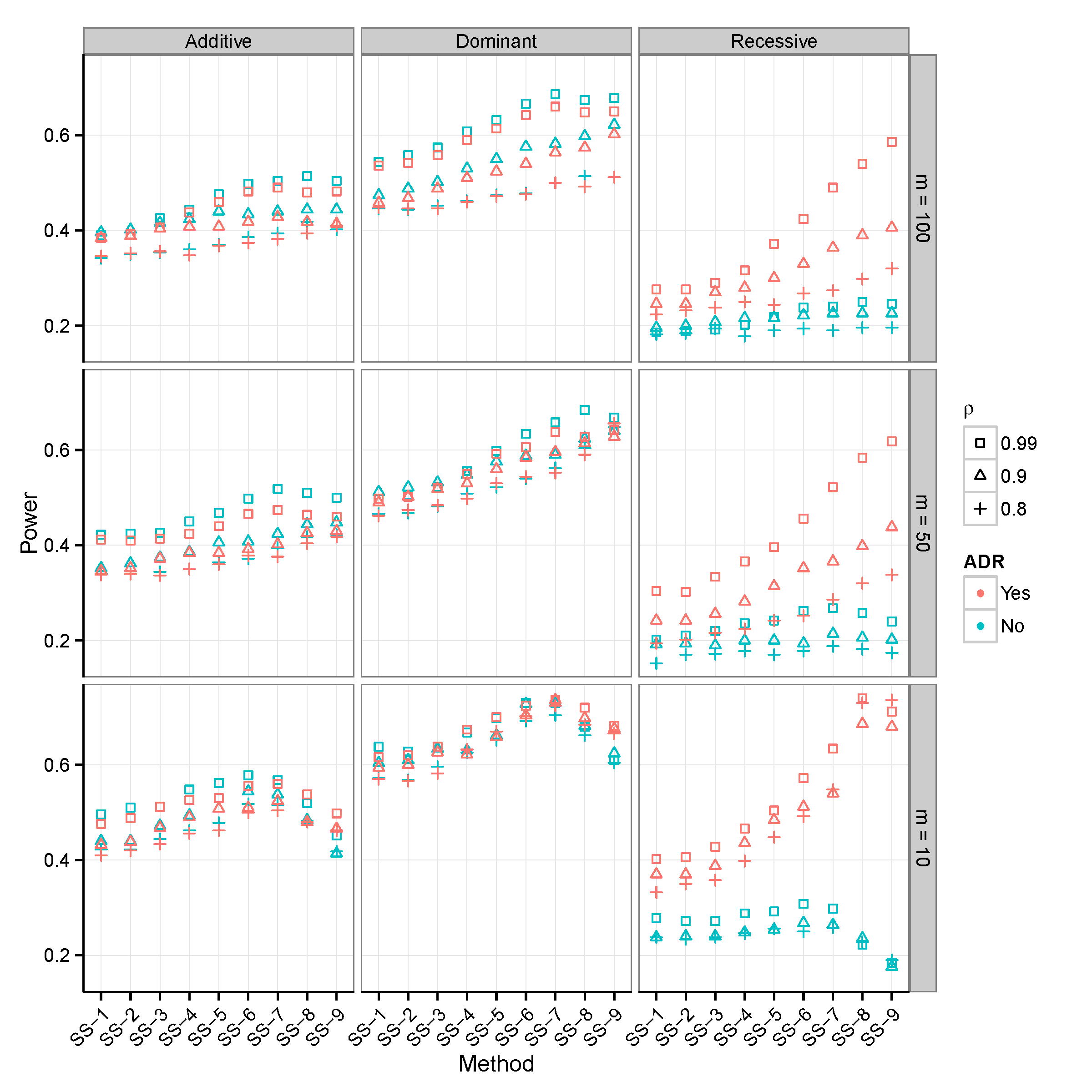

Supplement: Figure S4 — Empirical power of SS-T methods for the single causal variant scenario (k=1) under Experiment I as a function of the mode of inheritance (panels), the number of SNPs in the LD block (m), the polychoric correlation between the genotypes of SNPs in the LD block (ρ) and the ADR adjustment status. The causal allele frequency is p d=0.10 and the nominal type I error rate is α=0.05. See Figure S1 for background and abbreviations. (TIF) [file pone.0080540.s004.tif]

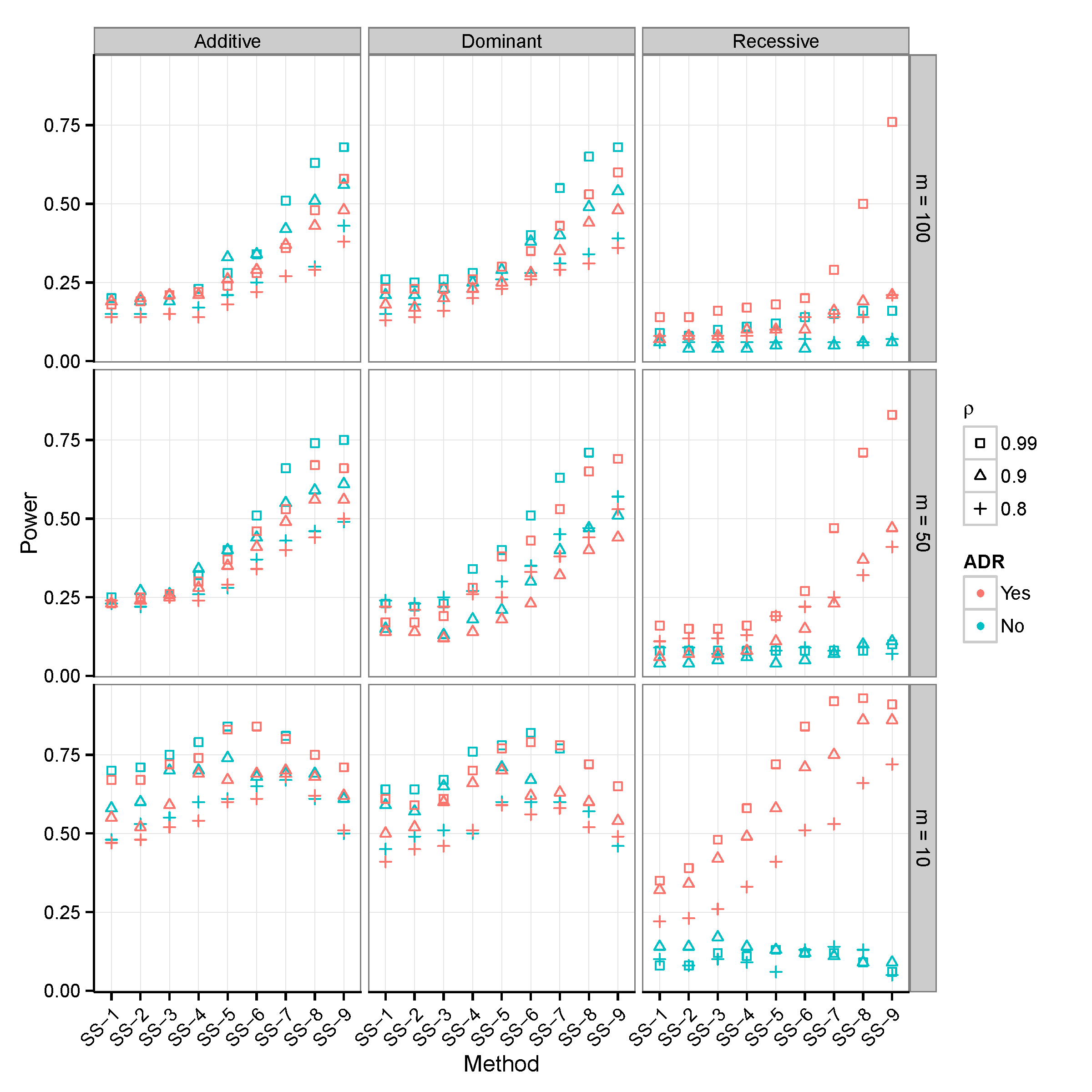

Supplement: Figure S5 — Empirical power of SS-T methods for the dual non-interacting causal variant scenario (k=2) under Experiment I as a function of the mode of inheritance (panels), the number of SNPs in the LD block (m), the polychoric correlation between the genotypes of SNPs in the LD block (ρ) and the ADR adjustment status. The causal allele frequency is p d=0.01 and the nominal type I error rate is α=0.05. See Figure S1 for background and abbreviations. (TIF) [file pone.0080540.s005.tif]

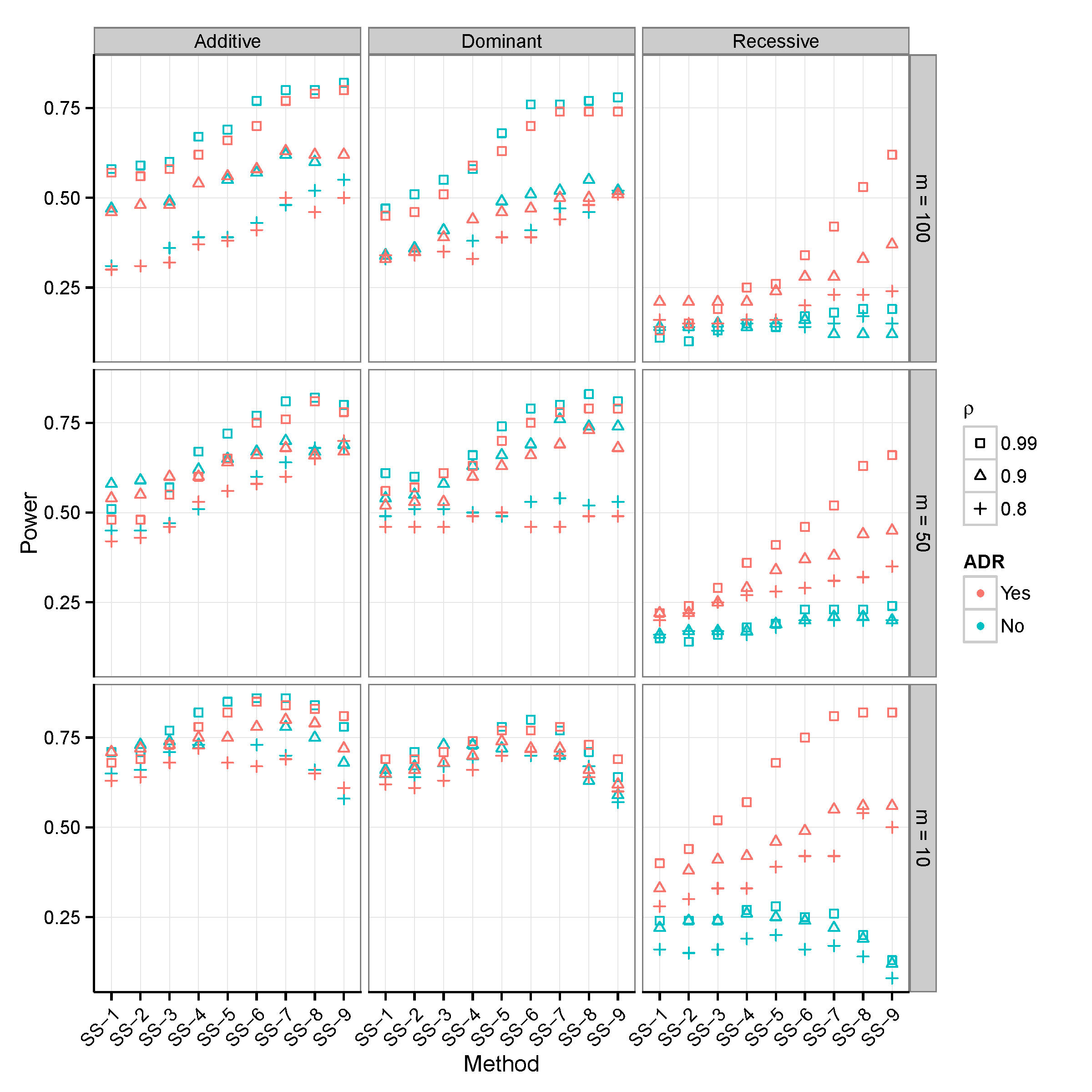

Supplement: Figure S6 — Empirical power of SS-T methods for the dual non-interacting causal variant scenario (k=2) under Experiment I as a function of the mode of inheritance (panels), the number of SNPs in the LD block (m), the polychoric correlation between the genotypes of SNPs in the LD block (ρ) and the ADR adjustment status. The causal allele frequency is p d=0.05 and the nominal type I error rate is α=0.05. See Figure S1 for background and abbreviations. (TIF) [file pone.0080540.s006.tif]

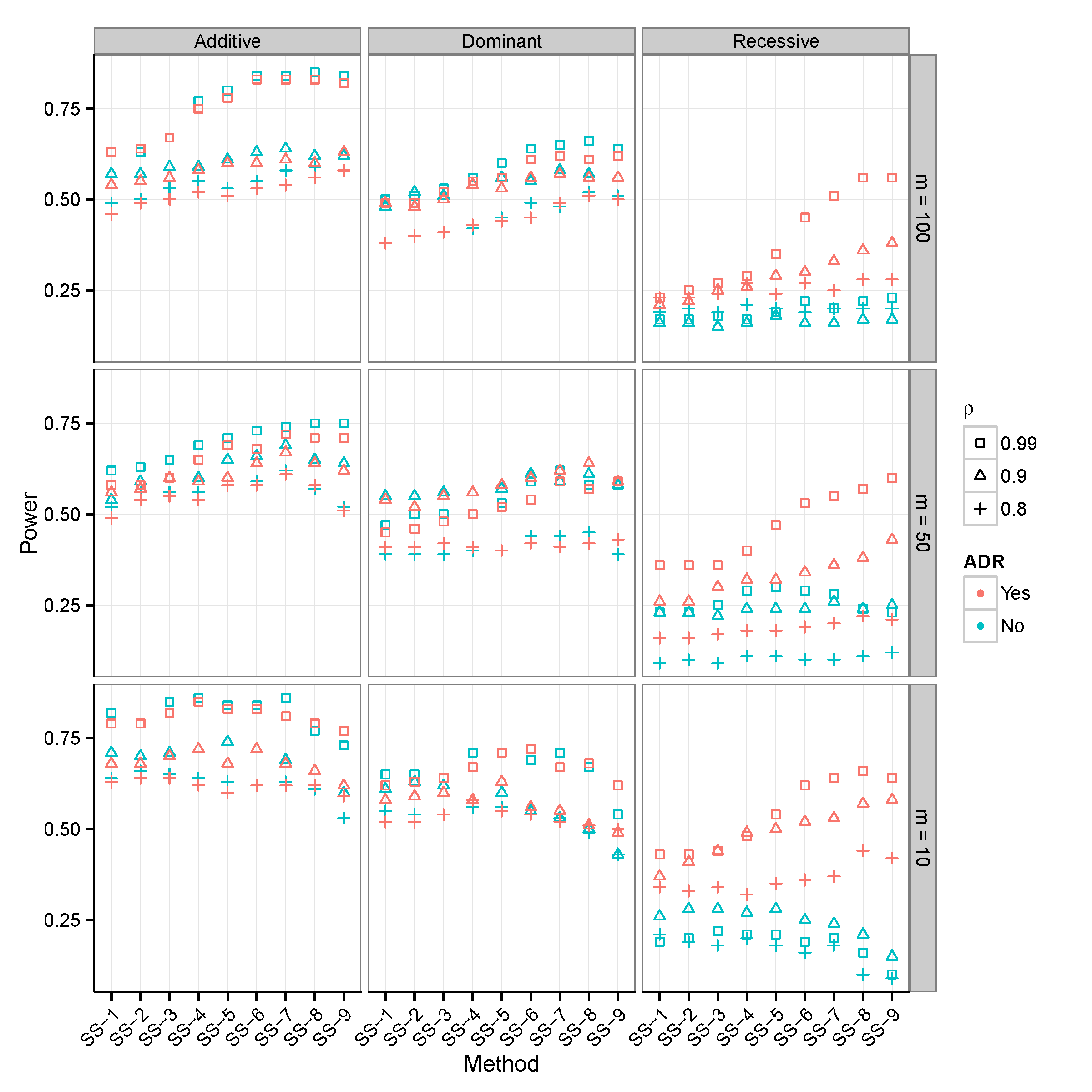

Supplement: Figure S7 — Empirical power of SS-T methods for the dual non-interacting causal variant scenario (k=2) under Experiment I as a function of the mode of inheritance (panels), the number of SNPs in the LD block (m), the polychoric correlation between the genotypes of SNPs in the LD block (ρ) and the ADR adjustment status. The causal allele frequency is p d=0.10 and the nominal type I error rate is α=0.05. See Figure S1 for background and abbreviations. (TIF) [file pone.0080540.s007.tif]

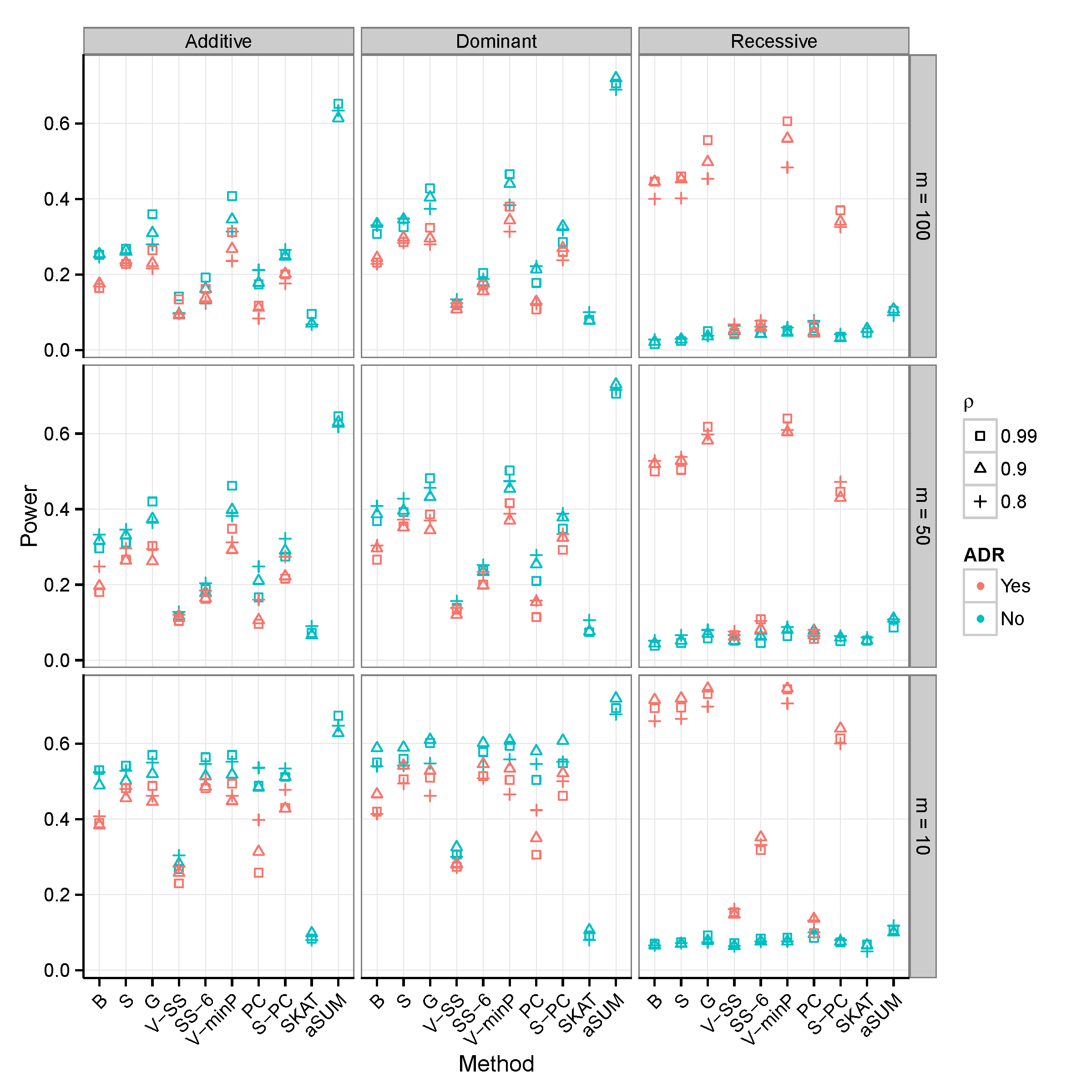

Supplement: Figure S8 — Empirical power of the main methods for the single causal variant scenario (k=1) under Experiment I as a function of the mode of inheritance (panels), the number of SNPs in the LD block (m), the polychoric correlation between the genotypes of SNPs in the LD block (ρ) and the ADR adjustment status. The causal allele frequency is p d=0.01 and the nominal type I error rate is α=0.05. See Figure S1 for background and abbreviations. (TIF) [file pone.0080540.s008.tif]

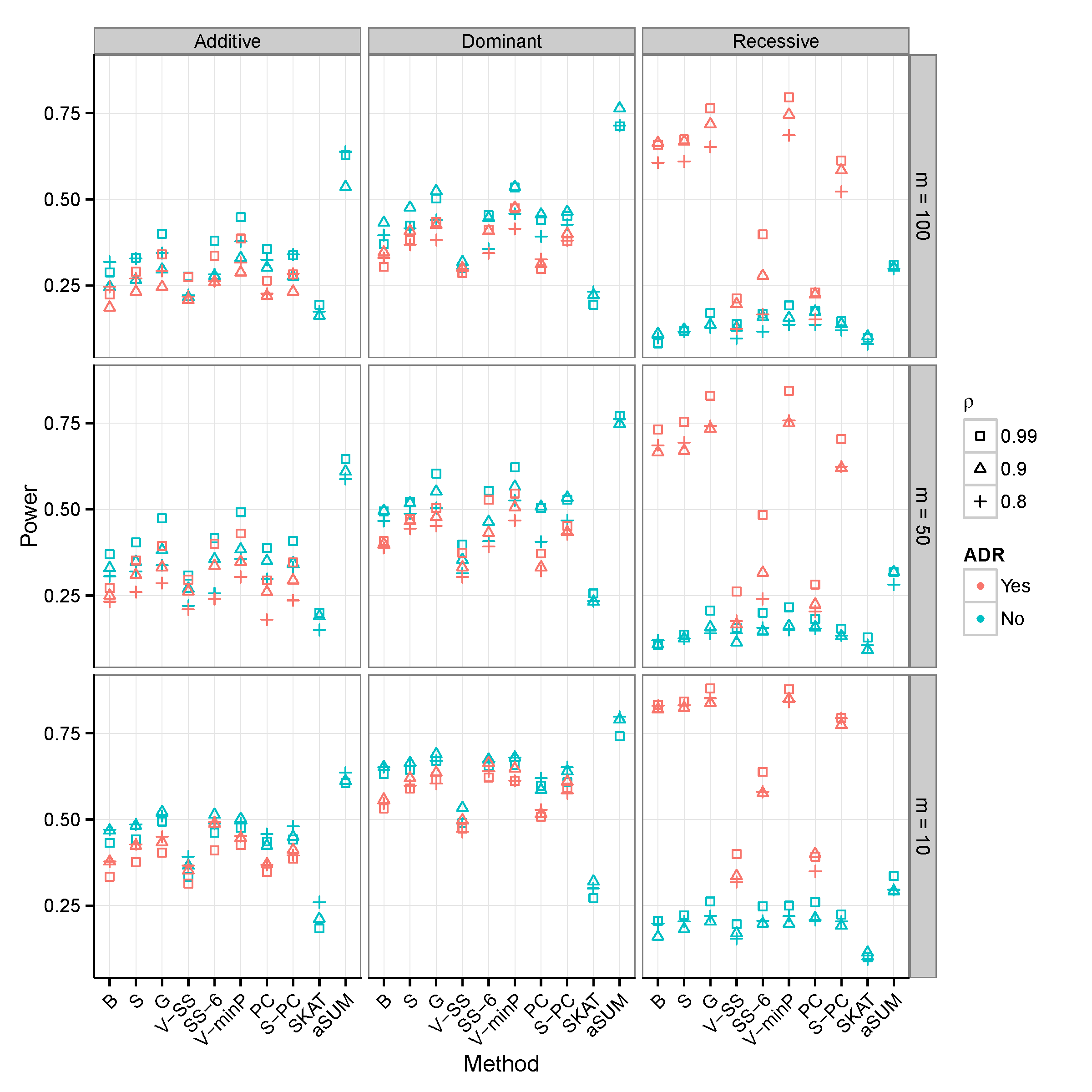

Supplement: Figure S9 — Empirical power of the main methods for the single causal variant scenario (k=1) under Experiment I as a function of the mode of inheritance (panels), the number of SNPs in the LD block (m), the polychoric correlation between the genotypes of SNPs in the LD block (ρ) and the ADR adjustment status. The causal allele frequency is p d=0.05 and the nominal type I error rate is α=0.05. See Figure S1 for background and abbreviations. (TIF) [file pone.0080540.s009.tif]

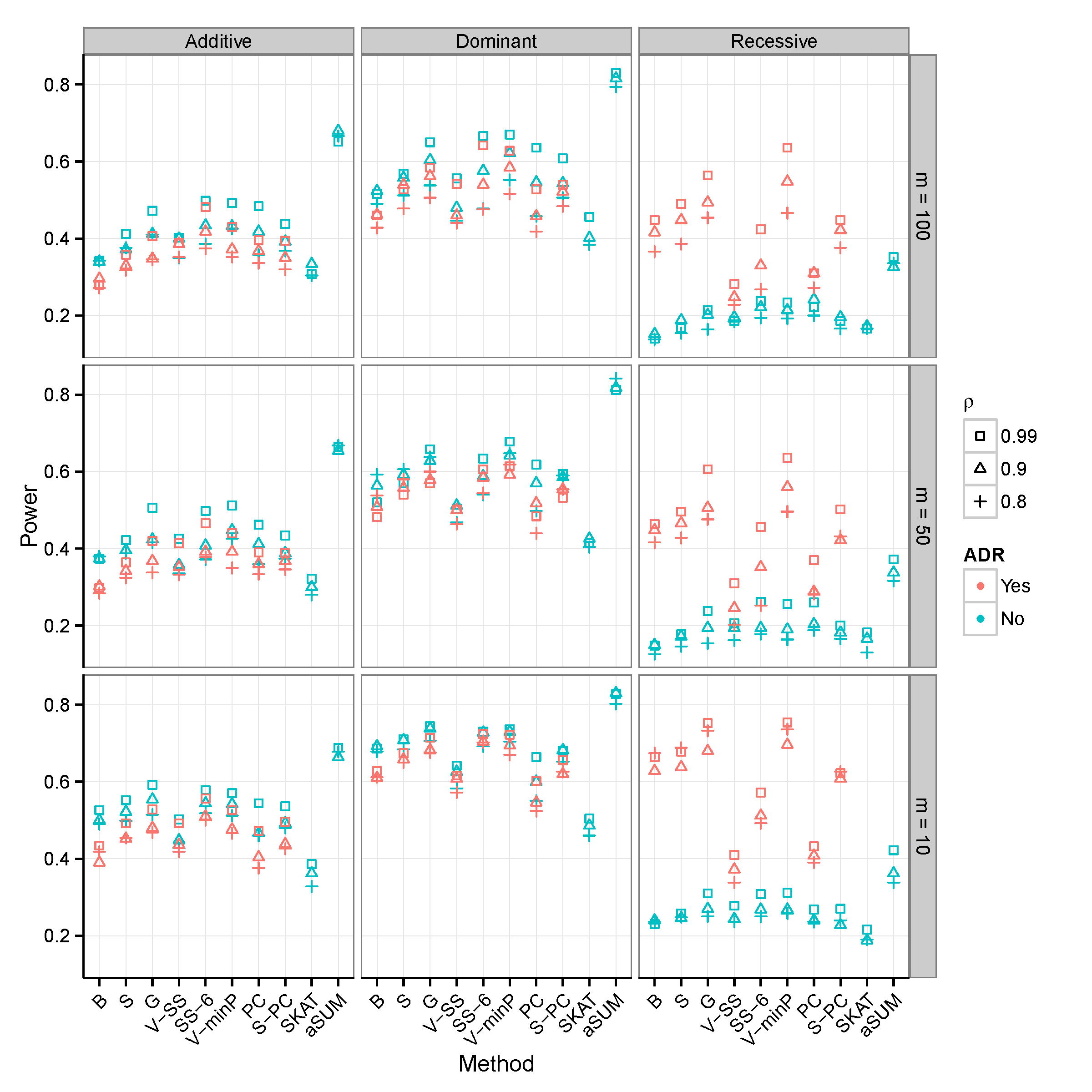

Supplement: Figure S10 — Empirical power of the main methods for the single causal variant scenario (k=1) under Experiment I as a function of the mode of inheritance (panels), the number of SNPs in the LD block (m), the polychoric correlation between the genotypes of SNPs in the LD block (ρ) and the ADR adjustment status. The causal allele frequency is p d=0.10 and the nominal type I error rate is α=0.05. See Figure S1 for background and abbreviations. (TIF) [file pone.0080540.s010.tif]

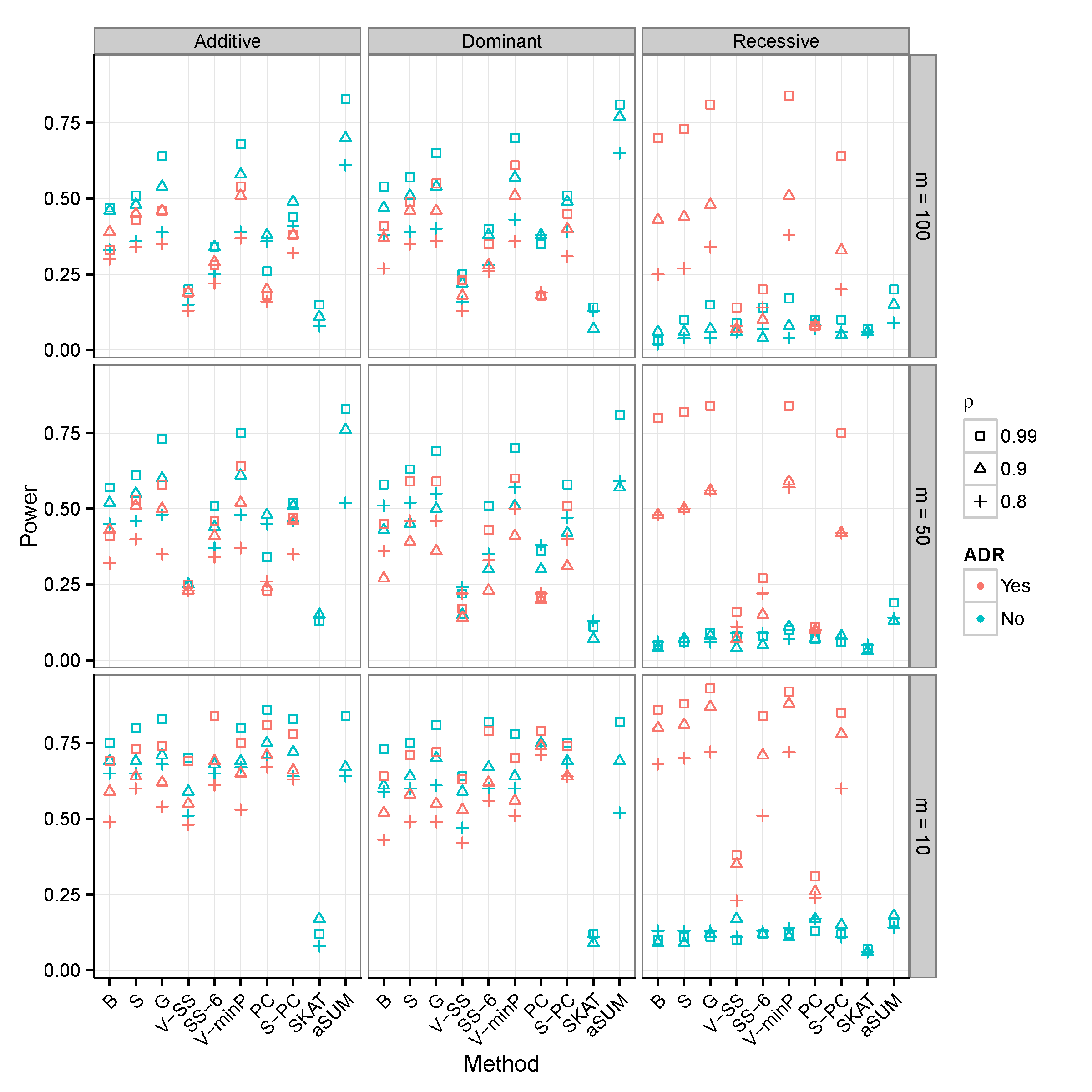

Supplement: Figure S11 — Empirical power of the main methods for the dual non-interacting causal variant scenario (k=2) under Experiment I as a function of the mode of inheritance (panels), the number of SNPs in the LD block (m), the polychoric correlation between the genotypes of SNPs in the LD block (ρ) and the ADR adjustment status. The causal allele frequency is p d=0.01 and the nominal type I error rate is α=0.05. See Figure S1 for background and abbreviations. (TIF) [file pone.0080540.s011.tif]

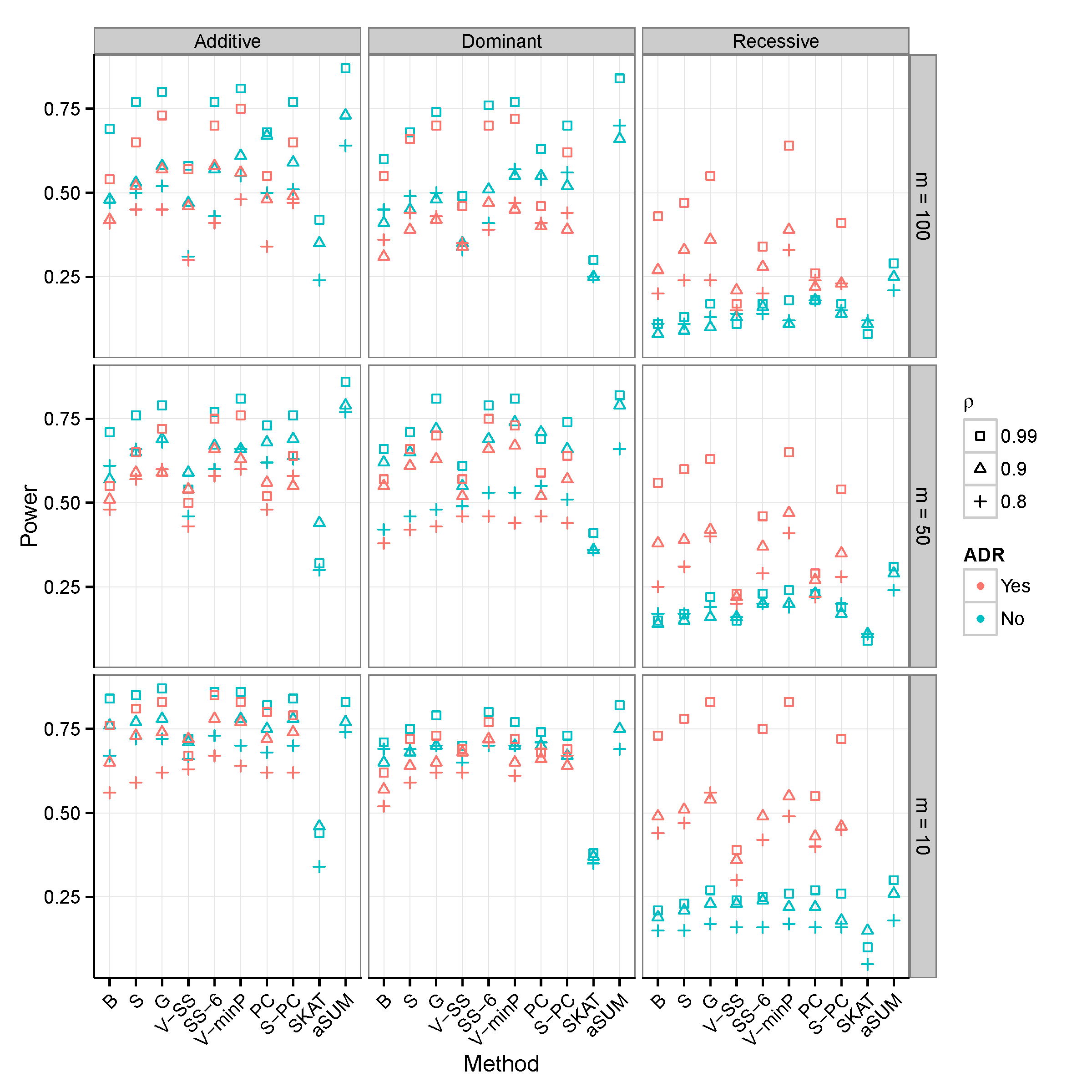

Supplement: Figure S12 — Empirical power of the main methods for the dual non-interacting causal variant scenario (k=2) under Experiment I as a function of the mode of inheritance (panels), the number of SNPs in the LD block (m), the polychoric correlation between the genotypes of SNPs in the LD block (ρ) and the ADR adjustment status. The causal allele frequency is p d=0.05 and the nominal type I error rate is α=0.05. See Figure S1 for background and abbreviations. (TIF) [file pone.0080540.s012.tif]

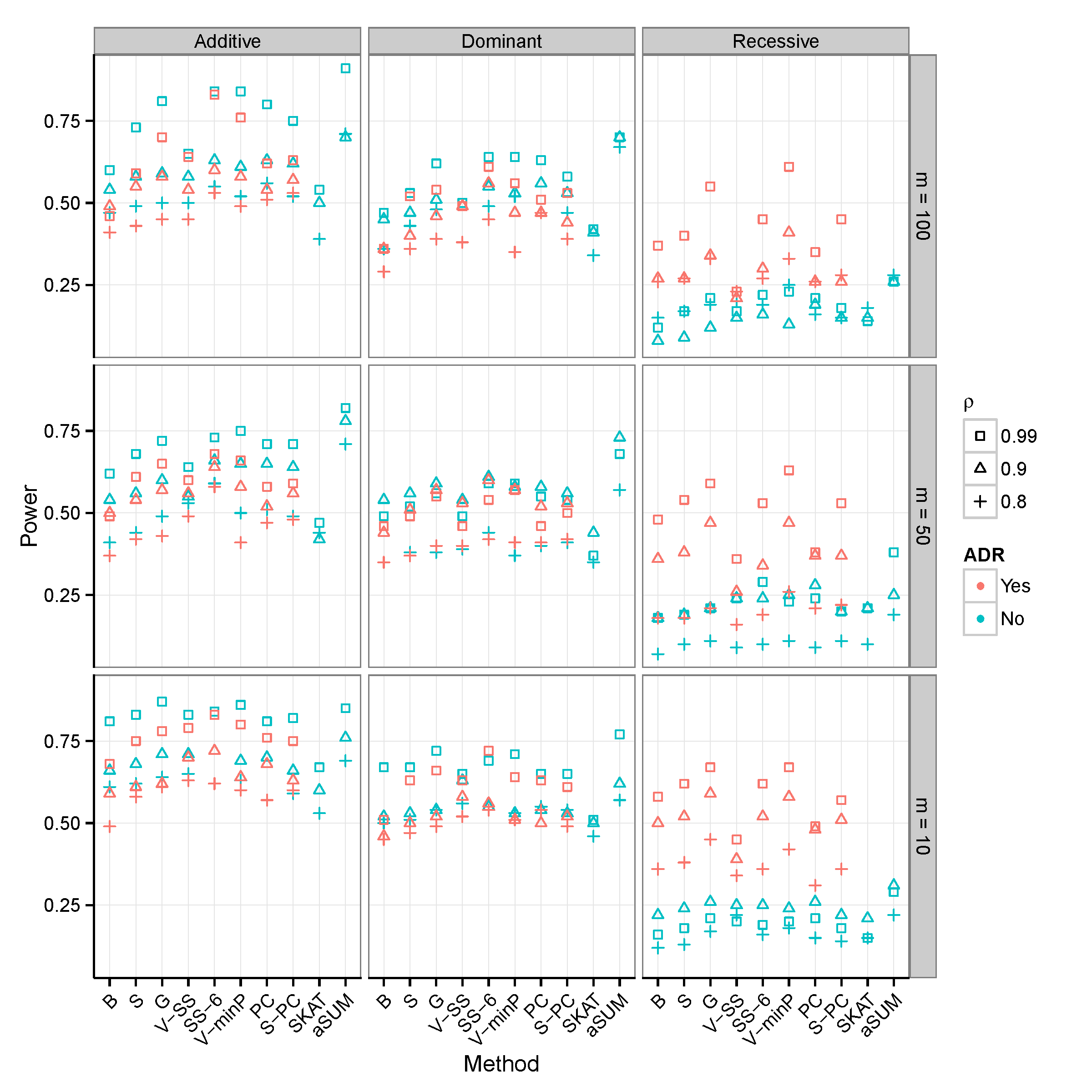

Supplement: Figure S13 — Empirical power of the main methods for the dual non-interacting causal variant scenario (k=2) under Experiment I as a function of the mode of inheritance (panels), the number of SNPs in the LD block (m), the polychoric correlation between the genotypes of SNPs in the LD block (ρ) and the ADR adjustment status. The causal allele frequency is p d=0.10 and the nominal type I error rate is α=0.05. See Figure S1 for background and abbreviations. (TIF) [file pone.0080540.s013.tif]
