# Supplementary material for: Association Testing Strategy for Data from Dense Marker Panels
Source: PLoS One. 2013 Nov 12;8(11):e80540. doi: 10.1371/journal.pone.0080540 (PMC3827222; doi:10.1371/journal.pone.0080540)
Supplement: methods S1 — Simulation of genotype-phenotype data. (DOC) [file pone.0080540.s014.doc]

**Methods S1. Simulation of genotype-phenotype data**

**Simulation of genotypic data**

We simulate the latent vector of size $m$, $Z=(Z_{1},\ldots,Z_{m})$, from a multivariate normal distribution assuming a zero mean and an exchangeable correlation structure, i.e. all non-diagonal elements of the correlation matrix have the same positive value $0<\rho<1$. We, then, simulate the RAFs of $m$ SNPs, denoted as $p=(p_{1},\ldots,p_{m})$, from a uniform distribution $U(0.01,\text{ }0.99)$. The $i^{\text{th}}$ entry of a genotype vector $G=(G_{1},\ldots,G_{m})$ is obtained by discretizing the $i^{\text{th}}$ element of the simulated latent vector. The discretization is achieved by using thresholds which are consistent with the assumption of Hardy-Weinberg Equilibrium in the population:

$$G_{i}=\left\{\begin{matrix} \begin{matrix} 0 \\ 1 \\ 2 \end{matrix} & \begin{matrix} \text{ if }\Phi(Z_{i})\leq(1-p_{i})^{2} \\ \text{ if }(1-p_{i})^{2}<\Phi(Z_{i}) \\ \text{ if }\Phi(Z_{i})>1-p_{i}^{2}, \end{matrix} \end{matrix}\right.\leq1-p_{i}^{2}$$

where $\Phi$ is the cumulative distribution function of the standard normal distribution. While in real data the relationship between genotypes might not be the one described by the multivariate normal distribution, this method i) captures the essential feature of co-occurring alleles and ii) is able to simulate all possible haplotypes in a block, unlike the small number which is available when using the small publicly available data sets.

**Simulation of phenotypic data under single causal variant scenario**

The phenotype of a subject is determined based on the penetrance(s) for the genotype(s) at the causal variant(s), as derived from the mode of inheritance and frequency. For a model containing a single causal variant associated with disease, the phenotype, $Y$, of a subject is determined as

$$Y=\left\{\begin{matrix} 1 & \text{ with probability }\Pr(D|G_{d})\text{ } \\ 0 & \text{ with probability }1-\Pr(D|G_{d}), \end{matrix}\right.$$

where $G_{d}$ is the genotype of the causal variant and $\Pr(D|G_{d})$ is the penetrance function. $\Pr(D|G_{d})$ is defined as

$$\Pr(D|G_{d})=\left\{\begin{matrix} \begin{matrix} f_{0} \\ R_{1}f_{0} \\ R_{2}f_{0} \end{matrix} & \begin{matrix} \text{ if }G_{d}=0\text{ } \\ \text{ if }G_{d}=1\text{ } \\ \text{ if }G_{d}=2\text{,} \end{matrix} \end{matrix}\right.$$

where $f_{0}$ is the baseline penetrance and $R_{1}$ and $R_{2}$ are the relative risks of heterozygote and risk allele homozygote compared with non-risk allele homozygote, respectively. $f_{0}$ is not an independent quantity and can be calculated as a function of other parameters as:

$$f_{0}=\frac{K}{p_{d}^{2}R_{2}+2p_{d}(1-p_{d})R_{1}+(1-p_{d})^{2}}\text{,}$$

with $K=\Pr(D)$ being the prevalence of the disease and $p_{d}$ being the causal allele frequency. Pairs of $R_{1}$ and $R_{2}$ used in single causal variant scenario under Experiment I and II are available in Tables S2 and 1, respectively.

**Simulation of phenotypic data under** $k$ **causal variant scenario (**$k>1$)

For the $k$ causal variant scenario **(**$k>1$), $Y$ is determined as

$$Y=\left\{\begin{matrix} 1 & \text{ with probability }\Pr(D|G_{d1},...,G_{dk}) \\ 0 & \text{ with probability }1-\Pr(D|G_{d1},...,G_{dk}), \end{matrix}\right.$$

where $\Pr(D|G_{d1},...,G_{dk})$ is the penetrance and $G_{d1},...,G_{dk}$ are the genotypes of the $k$ causal variants. For each mode of inheritance (additive, dominant and recessive), $\Pr(D|G_{d1},...,G_{dk})$ is defined as

$$\Pr(D|G_{d1},...,G_{dk})=\left\{\begin{matrix} \begin{matrix} f_{0}+\sum_{i=1}^{k} \delta G_{di} \\ f_{0}+\sum_{i=1}^{k} \delta I(G_{di}\geq1) \\ f_{0}+\sum_{i=1}^{k} \delta I(G_{di}=2) \end{matrix} & \begin{matrix} \text{for additive mode of inheritance} \\ \text{for dominant mode of inheritance} \\ \text{for recessive mode of inheritance} \end{matrix} \end{matrix}\right.$$

where $\delta\geq0$ is the effect size of any causal allele, i.e. effect sizes of the alleles at the $k$ causal variants are assumed to be identical. Effect sizes ($\delta$) used in Experiment I and II are given in Tables S3 and 2, respectively. $f_{0}$ is the baseline penetrance and, for simplicity, in our simulations we assumed that $f_{0}=K/k$.
