# Supplementary material for: Association Testing Strategy for Data from Dense Marker Panels
Source: PLoS One. 2013 Nov 12;8(11):e80540. doi: 10.1371/journal.pone.0080540 (PMC3827222; doi:10.1371/journal.pone.0080540)
Supplement: Table S1 — Settings used in Experiment I. (DOC) [file pone.0080540.s015.doc]

**Table S1.** Settings used in Experiment I

| Parameter name | Parameter | Number of causal variants ($k$) | | |
| --- | --- | --- | --- | --- |
|  |  | $k=0$ | $k=1$ | $k=2$ |
| Number of replicates | $n$ | 500 | 500 | 100 |
| Number of SNPs in LD block | $m$ | {10, 50, 100} | {10, 50, 100} | {10, 50, 100} |
| Causal allele frequency | $p_{d}$ | - | {0.01, 0.05, 0.1} | {0.01, 0.05, 0.1} |
| Polychoric correlation between SNPs in LD block | $\rho$ | {0.8, 0.9, 0.99} | {0.8, 0.9, 0.99} | {0.8, 0.9, 0.99} |
