# Supplementary material for: Association Testing Strategy for Data from Dense Marker Panels
Source: PLoS One. 2013 Nov 12;8(11):e80540. doi: 10.1371/journal.pone.0080540 (PMC3827222; doi:10.1371/journal.pone.0080540)
Supplement: Table S2 — Number of cases/controls (n), relative risk of heterozygote to non-risk allele homozygote (R1) and relative risk of risk allele homozygote to non-risk allele homozygote (R2) used at each simulation setting under the single causal variant scenario (k=1) of Experiment I. Within each cell, the settings are presented as n (R 1, R 2). (DOC) [file pone.0080540.s016.doc]

**Table S2. Number of cases/controls ($n$), relative risk of heterozygote to non-risk allele homozygote ($R_{1}$) and relative risk of risk allele homozygote to non-risk allele homozygote ($R_{2}$) used at each simulation setting under the single causal variant scenario ($k=1$) of Experiment I. Within each cell, the settings are presented as $n\text{ }(R_{1},\text{ }R_{2})$.**

|  | Genetic Model | | |
| --- | --- | --- | --- |
| CAF($p_{d}$) | Additive | Dominant | Recessive |
| 0.01 | 1,000 (2, 3) | 1,000 (2.1, 2.1) | 25,000 (1, 7) |
| 0.05 | 1,000 (1.4, 1.8) | 1,000 (1.5, 1.5) | 3,000 (1, 4) |
| 0.1 | 1,000 (1.3, 1.6) | 1,000 (1.4, 1.4) | 1,000 (1, 3) |
