# Supplementary material for: Association Testing Strategy for Data from Dense Marker Panels
Source: PLoS One. 2013 Nov 12;8(11):e80540. doi: 10.1371/journal.pone.0080540 (PMC3827222; doi:10.1371/journal.pone.0080540)
Supplement: Table S3 — Number of cases/controls (n) and effect size (δ) of any causal allele used at each simulation setting under the two non-interacting causal variant scenario (k=2) of Experiment I. Within each cell, the settings are presented as n (δ). (DOC) [file pone.0080540.s017.doc]

**Table S3. Number of cases/controls ($n$) and effect size ($\delta$) of any causal allele used at each simulation setting under the two non-interacting causal variant scenario ($k=2$) of Experiment I. Within each cell, the settings are presented as $n\text{ (}\delta\text{)}$.**

|  | Genetic Model | | |
| --- | --- | --- | --- |
| CAF() | Additive | Dominant | Recessive |
| 0.01 | 1,000 (0.0175) | 1,000 (0.0175) | 20,000 (0.125) |
| 0.05 | 1,000 (0.0075) | 1,000 (0.0075) | 1,000 (0.075) |
| 0.1 | 1,000 (0.005) | 1,000 (0.005) | 1,000 (0.025) |
